# Twelve Tips for Synergistic Delivery of Remote Advanced Pharmacy Practice Experience Rotations

**DOI:** 10.15694/mep.2020.000257.1

**Published:** 2020-11-18

**Authors:** Trisha Branan, W. Anthony Hawkins, Andrea Sikora Newsome, Christopher Bland, Susan Smith

**Affiliations:** 1 University of Georgia College of Pharmacy

**Keywords:** Experiential Education, Remote Learning, Health Professions Education, Educational Strategies

## Abstract

This article was migrated. The article was marked as recommended.

In response to the COVID-19 pandemic, many institutions changed their staffing models to incorporate remote work which created a need for pharmacy faculty and preceptors to also shift their rotation experience to a remote format. While initially this may be a daunting task, remote experiences have the potential to equip students with unique skillsets while offering a mutually beneficial effort towards patient care and/or other responsibilities. In addition, these remote experiences can offer students a more customized rotation and a behind the scenes look at the preceptor’s career. This article provides 12 tips for developing a remote learning experience.

## Introduction

Remote advanced pharmacy practice experience (APPE) rotations are powerful tools to maintain rotation site availability in the era of COVID-19 and have unique synergistic potential. In response to the COVID-19 pandemic, many institutions changed their staffing models to incorporate remote work and/or telehealth to mitigate virus transmission while still providing essential pharmacy patient care services. As a result, many student experiential policies limited rotation availability or onsite experiences. Pharmacy faculty and preceptors were asked to provide remote patient care or remote alternative learning experiences (e.g. academia rotation). While transitioning to an alternative APPE model can be challenging, the ability to provide a remote experience with flexible activities and timelines has unique appeal that can engage students in new skillsets while providing mutually beneficial effort on daily responsibilities and scholastic projects. Here, we outline 12 tips for successful delivery of a remote APPE. Although these tips are drawn from our experiences as preceptors of pharmacy student APPEs, many of us also serve as preceptors for medical student and pharmacy post-graduate resident experiences. The tips presented can be widely extrapolated to learners from many healthcare disciplines.

## Tip #1: Mentor and model for your students

Deliberate assignment of activities associated with professional development and scheduled discussion of the student’s professional aspirations is a mutually beneficial addition to any rotation. Assigned activities can include completion of personality inventories and other reflective exercises. Scheduled time purely focused on the discussion of the student’s global career aspirations can also support how other assigned activities support growth and development. Creating a sense of investment is discussed in Tip 6 but is certainly aided by deliberate mentorship by the preceptor and may be particularly advantageous given the millennial generation’s emphasis on understanding the “why” and the importance of a particular task ( Waljee, Chopra and Saint, 2020). Students are often quite observant to how a preceptor structures their day and their attitudes towards different activities. Scheduled reflection time to discuss how the preceptor prioritizes their day and aspects in their career can be fruitful areas of discussion. Ultimately, a remote rotation has the potential to be a highly productive and collaborative experience for both preceptor and student, and this experience largely depends on the development of a meaningful relationship. Indeed, the preceptor often learns new things from the student, like new technology platforms.

## Tip #2: Align assignments with current priorities

Despite the strong temptation when designing your remote rotation experience to create new activities that provide a comprehensive experience for your student, be intentional in assigning mutually beneficial activities. Quite simply, do not create more work for yourself as a result of having a student on a remote rotation. Evaluate your own to-do list and look for ways to break down larger projects into activities a student could engage in. Brainstorm on projects that have been on the backburner and evaluate if the student can help revitalize a project that has lost steam. Ultimately, assigning deliverables that are within the scope of activities you already need to accomplish is mutually beneficial as it helps you to accomplish your goal while providing purpose to the student in knowing that their assignment is truly important to you. This is a great way to save time, reduce “busy work”, and promote productivity for the preceptor and the student alike.

## Tip #3: Prioritize inclusivity

When allowed, include your student in everything you do. Have the student attend meetings with you, participate in conference calls, and engage in group emails. Being inclusive shows the student a degree of trust that will make them feel valued. This level of inclusion also provides a “behind the scenes” look that may provide a new point of view compared to their usual perspective. Since these interactions will occur in a remote environment, be sure to schedule some time to debrief with your student after the meeting or conference call. Provide your student an opportunity to describe what surprised them and what new perspective they gained. Be sure to share your reflections as well (e.g., Was this a typical meeting? How do you think that meeting could have been improved?). It is important for your student to be exposed to the good, bad, and ugly to gain a complete sense of a career, especially if they espouse interest.

## Tip #4: Expose the student to academic and non-direct patient care activities early

“What do you do when you aren’t teaching or with patients?” Many students will serve as preceptors with some even having a significant interest in academia as a profession in the future so exposing students to academic and non-direct patient care activities during the rotation is important. Without intentional discussion, students are unlikely to be oriented to the career path of academia or the many associated responsibilities that come with being a preceptor. Highlighting the myriad of the opportunities, obligations, and global construct including both promotion criteria and the academic triad (teaching, scholarship, and service) in addition to the other responsibilities outside of patient care activities should be core learning activities for the student. Describing time allocation to each of these areas is fundamental to the student’s understanding of the structure of the rotation and the preceptor’s entire job responsibilities. The unique mixture of high independence, lack of traditional structure, and requisite creativity make this a learning experience for which most students will require orientation.

## Tip #5: Intentionally assign deliverables with the student in mind

Deliberate thought regarding assigned work products can foster student engagement and mutually beneficial deliverables. Cognizance of a student’s prior experiences will provide insight into previously developed skills and ones requiring enhancement. Further, understanding a student’s skill level will aid in determining appropriate deadlines. Perhaps most notably with both patient care interventions and professional writing, deadlines should be balanced between allowing the student ample time to produce a meaningful first attempt while also being conservative enough knowing that the preceptor will likely need to provide substantial feedback.

As preceptors and academicians have multiple projects in various phases on top of daily responsibilities, introducing students to the concept of “having multiple irons in the fire” is a key rotation skill. Ensure students understand the scope of each task, associated skills, and approximate time allocation at the outset, and follow this up with deliberate discussion of assignment progress and frequent reassessment of priorities.

Assignments can and should be synergistic with one another, similar to faculty specialty areas. Preceptors should be intentional in stacking deadlines in a way that benefits the student with smaller tasks leading to larger tasks in the same topic area. In this manner, the preceptor can assess knowledge gaps and provide meaningful feedback that translates between projects. Further, for large assignments, help students learn basic project management skills akin to “Eat That Frog,” wherein the preceptor helps break down a big task into smaller bite size chunks while assigning the student to develop mini-deadlines for these smaller tasks ( Tracy, 2017).

## Tip #6: Actively engage students to invest and help design the experience

The lack of structure and need for creativity can be daunting when applied to assignments that may seem foreign, unimportant, or lacking purpose. Indeed, the expectation that students will work hard, in this new highly autonomous environment, to make meaningful contributions to assignments without being personally invested is likely unfounded. To increase investment, provide a selection of proposed assignments or professional obligations (e.g. committee meetings) to choose from and describe the importance of each and the associated expectations. Let them “choose their own adventure” by selecting which projects/meetings they see meaningful. Having students understand the importance of their contribution and appealing to their personal interests will provide motivation that their hard work will make an impact. You can further incentivize them with acknowledgements, opportunities to present their work, or authorship, where applicable. Further, seek the students input for what will make the experience most engaging.

## Tip #7: Empower the student

A remote rotation is an ideal setting to develop a student’s independent project management skills, and this skillset should be emphasized by the preceptor. In particular, use aspects of the “flipped classroom model” (e.g., outside readings and deliverables) while simultaneously assigning the student the responsibility to develop timelines, calendars, and task lists ( Wong *et al.*, 2014). This creates a sense of collaboration and ownership. Increasing independence also reduces time burden on meetings between the preceptor and student which can lead to meeting fatigue. Further, specific discussion of customized communication patterns is warranted (e.g., text vs. e-mail, daily summaries vs. ad hoc meetings, window of communication during day). As discussed in Tip #6, allowing choices in assigned activities based on personal interests gets ‘buy-in’ from the student. Putting the student in charge of project management and scheduling can add to this buy-in.

## Tip #8: Optimize electronic platforms for student engagement and shared project management

Because remote rotations inherently mean less face-time, optimizing technology to support a sense of engagement is essential. The emphasis here is thinking through the logistics of the rotation with an eye for both efficiency and connectivity. Though many permutations exist, an example set-up would include the use of a cloud-based sharing platform (e.g., Dropbox, Google Docs, Microsoft Teams) to house a shared calendar for the month, revisions and final drafts of all deliverables, and other logistics documents including the syllabus and an assignment list. Taking the time to create a list of deliverables with key directions, deadlines, and supporting materials can create added efficiency. Students can learn valuable skills in orchestrating an efficient meeting that are highly useful in any future career path. These can include using technology to garner meeting availability times, developing a meeting agenda, and sending calendar invitations with meeting links. Remote meeting software (e.g., Zoom) can also be leveraged to create additional learning opportunities (e.g., journal clubs, auditing classes, observing other faculty meetings).

## Tip #9: Create an environment of collaboration (connect your students)

In line with not creating new work for yourself and to be professionally generous, ask fellow colleagues for any tasks that may offer a unique learning opportunity for the student that will add to their experience. This ability to help out a colleague with a project of theirs also allows you to connect the student with new individuals and opportunities that may or may not be in your clinical specialty while also offloading some effort from you. There is growing evidence that adequate sampling of various options is important within many fields before ultimately specializing, so helping to expose students to areas outside of your field of specialty is optimal ( Epstein, 2019). Further, this type of task is an important learning opportunity for a student that mimics the professional world where often one is assigned a task in conjunction with another individual with whom they have never worked prior to that assignment (e.g., committee work in an organization).

## Tip #10: Be highly intentional with feedback

In a remote environment, where much of the communication occurs via electronic means, providing feedback remains a core part of the learning experience, especially given a lack of “face-time.” Being highly intentional with feedback can help ensure that it is provided and can be achieved in several ways: (1) put it on the calendar: schedule feedback (e.g., “Feedback Fridays”) during which the student can self-reflect and the preceptor can provide formative and summative feedback, (2) provide formal feedback for each assignment, and (3) use various formats for feedback (e.g., written and verbal, formal and informal). Also, a rotation experience is an optimal time for your students to practice delivering feedback in addition to receiving it. The student can provide formal feedback for the rotation experience and should also engage in evaluating other trainees who may be from different levels or professions, if applicable. This can easily be achieved if you have multiple trainees on the rotation, but can also be achieved by using technology to connect students across different APPEs (e.g., student on academia rotation can evaluate case presentations of student on clinical rotation) and across different class ranks (e.g., student on academic rotation can evaluate seminar presentation of P3 student).

## Tip #11: Exercise professional generosity: give credit freely

Recognition can have an important role in enhancing engagement and commitment and in helping your student recognize the value of their contributions. A good goal is to find a way to “give credit” for every activity assigned to your student so that they can easily see a direct and personal benefit from their work. “Credit” can be granted in several different ways: a line item to add to the student’s curriculum vitae, an acknowledgement in a published manuscript or in a formal presentation, or verbal recognition at a meeting of peers, to name a few. This professional generosity provides great value and sense of accomplishment to the student. In his book *Give and Take: Why Helping Others Drives Our Success*, Grant describes takers, matchers, and givers and explains that givers, who contribute to others without expecting anything in return, tend to achieve the most personal success in their professional careers ( Grant, 2013). We recommend embracing this sense of professional generosity by ensuring that each activity assigned to a student benefits them directly.

## Tip #12: Encourage the student to develop a holistic perspective. Acknowledge the greater purpose

Depending on the preceptor’s job responsibilities, scholarship may be a requirement and/or a metric for success. That does not mean it is simply a chore. There is a selection bias in academic positions to those that innately want to advance science, medicine, and the profession. In a non-academic environment, it is important to also strive to make advancements. Leave the profession better than you found it. This can be done in several capacities including maintaining a professional image, contributing to the advancement of the profession, and giving back. Model for the student how to be an agent of change. Serve as a mentor to a pharmacy student. Allow a pre-pharmacy student to shadow you. Make those impressions so profound that you inspire your student to continue the cycle of giving back ( Newsome, 2020).

**Table 1.** Application Examples for the 12 Tips

Tip	Examples
Mentor and model for your students.	• Assign deliverables such as updating a curriculum vitae, drafting letters of intent, developing teaching philosophies • Scheduled discussions related to student’s career aspirations and preceptor’s career path
Align assignments with current priorities.	• Student could perform literature search to guide a new research project or reformat a manuscript for submission to an alternative journal • Student could draft a new protocol or policy for your department
Prioritize inclusivity.	• Have the student attend meetings with you, participate in conference calls, engage in group emails
Expose the student to academic and non-direct patient care activities early.	• Schedule formal discussions with student related to the academic triad (teaching, scholarship, and service) and the promotion and tenure process • Discuss the other obligations of the preceptor outside of direct patient care (committee meetings, protocol/policy development, quality improvement projects, professional organization involvement)
Intentionally assign deliverables with the student in mind.	• Take an inventory of the student’s prior experiences and skills at the start of the rotation to gauge entry level abilities • Be intentional in stacking deadlines to benefit the student (develop a short slide presentation on a topic before drafting a review article on the same topic) • Break down a larger project into smaller tasks with mini-deadlines (instead of assigning the task of writing a manuscript, consider assigning sequential tasks of first writing the methods, then write the results)
Actively engage students to invest and help design the experience.	• Allow the student to select which meetings to attend from a list of all committee or professional organization meetings that occur during the rotation • Provide the student with several manuscripts or projects, each in different phases requiring different levels of attention, and describe the importance of the topics to the institution, profession, or patients and allow them to choose which they would like to complete
Empower the student.	• Assign the student the responsibility to develop their rotation calendar, timelines, and task lists • Help the student develop lifelong learning skills through independent topic research prior to discussion with the preceptor
Optimize electronic platforms for student engagement and shared project management.	• Implement the use of a cloud-based sharing platform to house shared documents like calendars, project documents and revisions, rotation syllabus • Help the student develop efficient meeting management skills like polling attendees for availability times, creating a meeting agenda, and sending calendar invitations with meeting links
Create an environment of collaboration (connect your students).	• Ask colleagues for any tasks or projects that lend themselves to a learning opportunity for the student • Connect your student with other collaborators to expand their exposure to others outside your specialty area
Be highly intentional with feedback.	• Schedule feedback sessions (formal and informal, written and verbal) • Ask your students to provide feedback on the rotation experience or your precepting to help the student practice giving feedback • If available, ask students to provide feedback to other trainees
Exercise professional generosity: give credit freely/liberally.	• Find a way to give the student credit for every assigned activity (a line item to add to the student’s curriculum vitae, an acknowledgement in a published manuscript or in a formal presentation, or verbal recognition at a meeting of peers, etc.)
Encourage the student to develop a holistic perspective. Acknowledge the greater purpose.	• Discuss with the student the importance of giving back to the profession and investing in others • Provide examples of ways to advance the profession (original research, review articles, serving as a preceptor, involvement in professional organizations, mentoring others)

## Conclusion

Remote APPEs, while not face-to-face, offer a rewarding opportunity for both the student and the preceptor. It is important to balance the productivity of the rotation and provision of a customized and personal experience. Sampling of most aspects of the preceptor’s work provides the student with broad, honest exposure within the profession, often a mystery to most students. Further focusing on common interests within current projects of both the student and the preceptor will allow for a mutually beneficial experience leading often to new information being gained by both parties and synergistic productivity. Thoughtful feedback and intentional generosity provided throughout the rotation will lead to a deep, meaningful experience for students regardless of the remote method.

## Take Home Messages

• As a response to the COVID-19 pandemic, many healthcare institutions have incoporated remote work for patient care or other responsibilities, which has created a need for pharmacy faculty and preceptors to shift their experiential education rotations to a remote format.
• Remote education has the potential to create a mutally beneficial experience for both the student and preceptor.
• Students are able to have a more customized rotation experience, gain exposure into all aspects of the profession, and develop unique skills.
• Preceptors and faculty can find synergistic opportuntities to involve students in patient care responsibilities and scholarly projects.

## Notes On Contributors

**Trisha N. Branan**, Pharm.D., BCCCP is a clinical associate professor at the University of Georgia College of Pharmacy and an associate professor at the Medical College of Georgia at Augusta University. ORCID ID: https://orcid.org/0000-0002-7444-7417

**W. Anthony Hawkins**, Pharm.D., BCCCP is a clinical associate professor at the University of Georgia College of Pharmacy and an assistant professor at the Medical College of Georgia at Augusta University. ORCID ID: https://orcid.org/0000-0002-4083-7919

**Andrea Sikora Newsome**, Pharm.D., BCPS, BCCCP is a clinical assistant professor at the University of Georgia College of Pharmacy. ORCID ID: https://orcid.org/0000-0003-2020-0571

**Christopher M. Bland**, Pharm.D., FCCP, FIDSA, BCPS is a clinical associate professor at the University of Georgia College of Pharmacy. ORCID ID: https://orcid.org/0000-0001-8806-4583

**Susan E. Smith**, Pharm.D., BCPS, BCCCP is a clinical assistant professor at the University of Georgia College of Pharmacy and an assistant professor at the Medical College of Georgia at Augusta University. ORCID ID: https://orcid.org/0000-0002-5171-8405

## References

[ref1] Epstein D. J. (2019) Range : why generalists triumph in a specialized world. New York: Riverhead Books.

[ref2] Grant A. M. (2013) Give and take : a revolutionary approach to success. New York, N.Y.: Viking.

[ref3] Newsome A. S. (2020) Pay it forward. Am J Health Syst Pharm. 77(14), pp.1166–1168. 10.1093/ajhp/zxaa125 32415835 PMC8012882

[ref4] Tracy B. (2017) Eat that frog! : 21 great ways to stop procrastinating and get more done in less time. Third edition. edn. Oakland: Berrett-Koehler Publishers, Inc., A BK Life Book.

[ref5] Waljee J. F. Chopra V. and Saint S. (2020) Mentoring Millennials. JAMA. 323(17), pp.1716–1717. 10.1001/jama.2020.3085 32369152

[ref6] Wong T. H. Ip E. J. Lopes I. and Rajagopalan V. (2014) Pharmacy students’ performance and perceptions in a flipped teaching pilot on cardiac arrhythmias. Am J Pharm Educ. 78(10), p.185. 10.5688/ajpe7810185 25657372 PMC4315207

