# Supplementary material for: Twelve Tips for Synergistic Delivery of Remote Advanced Pharmacy Practice Experience Rotations
Source: MedEdPublish (2016). 2020 Nov 18;9:257. [Version 1] doi: 10.15694/mep.2020.000257.1 (PMC10697600; doi:10.15694/mep.2020.000257.1)
Supplement: Peer review history

# Reviewer report: Reviewer response for version 1

**DOI:** 10.21956/mep.20203.r31273

**Published:** 2021-02-06

**Authors:** Ken Masters

**Affiliations:** 1 Sultan Qaboos University

This review has been migrated. The reviewer awarded 3 stars out of 5 The paper is an interesting Twelve Tips article dealing with the synergistic delivery of remote advanced pharmacy practice experience rotations. It is a useful paper giving practical tips – and the Table with practical examples is a nice summary.There are, however, a few issues that should be addressed.• It would be useful if the authors could include a (perhaps abbreviated) listing of the tips in the Abstract. This is useful because many readers read the Abstract, and then decide whether the paper is worth reading or not. Listing the tips in the abstract will give them a clear indication of the content and value of the paper.• I can see why some people may find Tip 2 a little controversial. Readers need to realise that the tip’s value lies in recognising the fact that busy faculty are trying to cover so much, that not everything can be done properly and that, given that there is such a wide range of activities, assigning those that are mutually beneficial, will have a positive outcome. It is well-balanced by Tip 5. Perhaps, though, a line could be added to Tip 2, that the faculty should simultaneously guard against simple expediency and abuse of their powerful position, relative to the students’. • The paper is a little thin on drawing from the literature, and it would be useful if some of the material could be supported from the literature. (Simultaneously, however, I realise that the novel situation means that specific literature may be scarce).• There are a few other issues raised by the other reviewers, so I will not deal with them also.So, overall, a useful paper, but one in which some extra work would make more valuable. I look forward to version 2 in which the issues are addressed.

Reviewer Expertise:

NA

No decision status is available

# Reviewer report: Reviewer response for version 1

**DOI:** 10.21956/mep.20203.r31274

**Published:** 2020-12-01

**Authors:** Mojdeh Heavner

This review has been migrated. The reviewer awarded 5 stars out of 5 Thank you for the opportunity to comment on this article. Overall, the authors have provided a comprehensive, thoughtful guidance on how to improve the remote delivery of advanced practice experience rotations. I enjoyed reading this article and will plan to share it with my colleagues as a good example for how to create a more engaging experience for students on remote rotations. We are encountering unchartered territory with the pandemic and the move to more remote activities than we have done in the past. Schools have shifted teaching and learning strategies to adapt to this new environment, but with little data or past experience to use as examples. In reading this article and the 12 tips, I have learned some new strategies and recognized somethings that perhaps I was already doing but did not realize were happening intentionally. It is of the utmost importance to share our experiences in these times, and I applaud the authors for taking on this task. I have listed a few more detailed observations and comments below:-I believe that all of these tips can be applied to onsite, in-person rotations as well. It is a silver lining of the pandemic, that we have been forced to think about our world a little differently.-Tip #2: It appears this tip is more controversial than the authors intended. I agree 100% with the intent and how it is delivered as well. I think of my learners as an extension of me. Therefore, what tasks can they take on that "help" me conduct my job? I don't find anything wrong with this approach, given that the goal is to train learners to become us one day. It is key, however, to give students opportunities that will also be of benefit to them. In this regard, I think it is important to connect Tip #2 with Tip #5 and Tip #6. In engaging my students in my "to do" list, I often assign the patient care tasks (e.g., write the vancomycin kinetics note, write the anticoagulation monitoring note, communicate with the team via the hospital messaging system what changes need to be made to pharmacotherapy). However, with the activities and opportunities not directly impacting patient care, I will often provide the student with a choice (i.e., help review this paper OR data collect for this project).-Tip #3: This is an excellent point that I think we frequently forget about. It is probably not necessary to include students in all conference calls, but even involving them in some of these meetings will help them gain a greater sense of "what it is their preceptor does all day." I have been hesitant to include students in my meetings but will consider this now seeing that others are doing more of it. -I also love Tip #9. This is an example of something many of us are doing but don't realize that we are. It is helpful to be aware of it and connect students and express that the intent is for them to expand their professional network and collaboration opportunities. The authors, all faculty at University of Georgia, have an excellent track record of conducting research with heavy student involvement, so it makes sense to have this recommendation come from the UGA group. -Is Tip #10 is not unique to the online environment but I can see how the authors expanded on the importance of intentional feedback in the remote setting, by adding opportunities to have students evaluate one another and even adding a layered learning model. -I suggest the authors add a few statements about how all of these tips can be applied in a more traditional rotation experience as well, and that being even more compelled to ensure engagement in the remote setting has prompted us to reevaluate our experiential teaching strategies.

Reviewer Expertise:

NA

No decision status is available

# Reviewer report: Reviewer response for version 1

**DOI:** 10.21956/mep.20203.r31275

**Published:** 2020-11-20

**Authors:** Amjad Qandil

This review has been migrated. The reviewer awarded 3 stars out of 5 I have enjoyed reading is article “Twelve Tips for Synergistic Delivery of Remote Advanced Pharmacy Practice Experience Rotations”.The reviewers have the following comments:1. Most of the tips (Tips #1, 3-6, 11-12) provided in this manuscript will enrich APPE and other health professions rotations regardless of the format. What is missing is a clear narrative on how do these tips have an added value in the online environment, and/or how the online environment can facilitate the application of these tips in a way that was not possible/less effective in the traditional onsite setting. For example, Tip #3 is also applicable for onsite rotation. It is useful to provide a narrative on how these reflection sessions can be conducted online and how, if any, the online format can add value or facilitate this practice. It can be argued that this tip is extremely facilitated by the online format. Where students could have been considered a burden when attending all face-to-face meetings in which their preceptors are involved, the online format reducer barriers of physical room crowdedness or availability of suitably sized conference rooms. 2. Tip #2 Seems to be a “questionable” one. Does the authors suggest that the preceptors own priorities are more important than the objectives of the rotations? There is apparent contradiction with tip #5. It can be argued that this tip needs better articulation.3. Tip #7. Although the Flipped classroom teaching methodology has gained more recognition and traction with the transition to online remote education due to COVID-19, it has been used in face-to-face learning. It should be argued that it should be a main stay in all rotations regarding of the format.4. For Tips #8 and 9, it will be useful to mention that the experience gained form these two tip can enrich onsite rotations also.5. There was no clear mention if the authors are having hybrid format rotation in mind, post COVID-19 may be. May be adding this possibility to the mix will improve the utility of the manuscript. 6. Finally, it might be useful that preceptors should coordinate to safeguard against redundancy of over-including some of these tips in their rotations. For example, Tip #1, in how many rotations should a student be asked to update their CVs or draft letters of intent.Wish the authors the best of luck.

Reviewer Expertise:

NA

No decision status is available
